# Supplementary material for: Body image disturbance and associated eating disorder and body dysmorphic disorder pathology in gay and heterosexual men: A systematic analyses of cognitive, affective, behavioral und perceptual aspects
Source: PLoS One. 2022 Dec 6;17(12):e0278558. doi: 10.1371/journal.pone.0278558 (PMC9725123; doi:10.1371/journal.pone.0278558)
Supplement: Attachment — Submitted filename: Response to Reviewers_PONE-D-21-24159.docx [file pone.0278558.s002.docx]

Revised response to Editor and Reviewers

Manuscript: *Body image disturbance and associated eating disorder and body dysmorphic disorder pathology in gay and heterosexual men:* *A systematic analyses of cognitive, affective, behavioral und perceptual aspects* (PONE-D-21-24159)

Dear Editor and Reviewer,

thank you for the opportunity to respond to any remaining comments. We did our best to respond to your remarks. Please find detailed comments on your remarks and their implementation in the manuscript underneath each notation. The page references refer to the version of our manuscript with track changes.

Editor’s comments to the Author:

1. Please review your reference list to ensure that it is complete and correct. If you have cited papers that have been retracted, please include the rationale for doing so in the manuscript text, or remove these references and replace them with relevant current references. Any changes to the reference list should be mentioned in the rebuttal letter that accompanies your revised manuscript. If you need to cite a retracted article, indicate the article’s retracted status in the References list and also include a citation and full reference for the retraction notice.

*We thoroughly checked our reference list for any papers that had been retracted. Fortunately, that was not the case for any manuscript that we cited.*

Reviewers' comments to the Author:

Reviewer #3: 


1. To continue the commentary on gender and sex differentiation in this paper, I appreciate the consideration that the authors have already paid, and challenges related to language differences across English and German. I would also still encourage the authors to avoid the term “male” when possible, even as an adjective, due to specific language differentiation in English. Therefore, I would encourage the removal of the sentence: “Also, we use male as the adjective of man and not to differentiate between gender and sex” and instead rephrase to avoid using male. (E.g., “…Buhlmann and colleagues found that 27% of MEN IN THE STUDY reported at least one body-related concern…” as opposed to “…Buhlmann and colleagues found that 27% of MALE PARTICIPANTS reported at least one body-related concern…”

*Thank you for your valuable suggestion. We gladly accept your suggestion (“A representative German study by Buhlmann and colleagues found that 27% of men in the study reported at least one body-related concern”, p. 4, l. 67) and also deleted the term “male” throughout the rest of the manuscript (with one exception, see comment no. 6), e.g. “The BAS-2 has been validated for the use with sexual minority men and women”* *as opposed to “The BAS-2 has been validated for the use with a male and female sexual minority sample”, p. 16, l. 328. Accordingly, we removed the sentence: “we use male as the adjective of man and not to differentiate between gender and sex” from our manuscript.*

2. “Extensive, yet narrow” is a confusing phrase that the authors use multiple times throughout the paper now. Narrow is an antonym of extensive and therefore, this is somewhat oblique in meaning. I would encourage the authors to use a different phrase. If the literature is quantitively large, but narrow in scope, that is the phrase I would use.

*You are right, thanks for making us aware of that and suggesting a more specific term. We were happy to exchange the term throughout the manuscript (e.g. p. 3, l. 23)*.


3. On page 4, line 81-82, the authors state that no studies have compared drive for leanness between gay and heterosexual men. This is not strictly true, although their analysis do make interpretation difficult. See:
Strübel, J., & Petrie, T. A. (2019). Appearance and performance enhancing drug usage and psychological well-being in gay and heterosexual men. Psychology & Sexuality, 10(2), 132–148. <https://doi.org/10.1080/19419899.2019.1574879>

*Thanks for making us aware of this study. We included it in our manuscript (“Concerning the drive for a lean body (i.e., a trained, tight physique), only one comparative study exists, indicating that gay men have a stronger drive for leanness than heterosexual men”, p. 6, l. 104).*

4. I have a bit of a quibble with the assertion on page 4, line 85. Exercise behavior varies widely in terms of its goals and outcomes. Patients with anorexia who are solely focused on thinness concerns often engage in exercise to lose weight or avoid weight gain. In that context, exercise behavior is not geared towards gaining muscle, but rather losing fat. Thus the phrase on line 85 conflates exercise as only muscle-related when this is, in fact, not the *case.*

*You are right, this statement might be too restrictive. We changed the sentence to: “In contrast to the cognitive-affective component of body image, only a small number of studies have focused on the behavioral component of body image disturbance in gay men, and if so, predominantly on exercise behavior.” (p. 6, l. 108)*

5. I’m somewhat surprised that nowhere in the introduction is a mention of muscle dysmorphia. BDD broadly is associated with body image concerns, but when those concerns are narrowed to muscularity, individuals are often diagnosed with muscle dysmorphia. This does not need to be a main point of the introduction, but I think it’s worth a mention.

*That is a valid point. We added the following section to our introduction: “**Regarding muscle dysmorphia, a subtype of BDD characterized by a pathological concern about one’s muscularity, evidence is equally limited. For instance, in a validation study of the Muscle Dysmorphic Disorder Inventory (MDDDI [39]), sexual minority men reported qualitatively higher MDDI total scores than heterosexual men [40]. Furthermore, in a recent Italian study [41] nearly 9% of sexual minority men exhibited a high risk of being diagnosed with muscle dysmorphia, which was again, higher than that found in heterosexual samples.” (p. 8, l. 154)*

6. I find the term “male gay community” to be somewhat of a misnomer. The items on the Identification and Involvement With the Gay Community Scale actually explicitly include items mentioning gay, lesbian, and bisexual individuals, and can therefore be of varying gender identities. I would encourage the authors to remove the descriptor “male” from describing this scale. “Gay” can often be used as a blanket term for individuals that are not heterosexual, but I would also encourage the authors to refer to this construct as “sexual minority community” to fully describe its facets.

*Thank you for bringing this up. The Identification and Involvement with the Gay Community Scale (IGCS) does mention reading gay or lesbian oriented paper or magazine, or attending gay or lesbian organizational activities (presumably as those usually address the LGBTQ+ community as a whole, and not only the gay community), but overall, the scale is explicitly “designed to measure involvement with and perceived closeness to the gay community among men who have sex with men” and not within the lesbian or any other sexual minority community (Vanable et al., 2011). This was of utterly importance to us, as the gay and the lesbian communities seem to have opposite effects on community member’s body image. While affiliation with the lesbian community seems to decrease body image concerns (e.g. Henn et al., 2019), affiliation with the gay community seems to increase body image concerns (e.g. Pachankis et al., 2020; Tylka et al., 2012). As the term “gay” can be used for all genders (as you already mentioned), we wanted to avoid ambiguousness by adding the word “male”. In doing so, we followed the APA guidelines for bias free language, that state: “The terms* gay *as an adjective and* gay person *as a noun have been used to refer to both males and females. However, these terms may be ambiguous in reference [..]. Thus, it is preferable to use* gay *or* gay person *only when prior reference has specified the gender composition of this term”. And further: “Lesbian and gay male are preferred to the word homosexual when used as an adjective referring to specific persons or groups, and the terms lesbians and gay men are preferred to homosexuals used as nouns when referring to specific persons or groups” (*[*https://www.apa.org/pi/lgbt/resources/language*](https://www.apa.org/pi/lgbt/resources/language)*). As a compromise, we deleted the term* male *in the term* male gay community *throughout the manuscript except when describing the scale to specify the gender composition of this term, as requested by the APA. Secondly, we tried to clearly differentiate between the gay and the lesbian community by describing: “Besides discrimination, it is assumed that pressure from within the gay community to be attractive and muscular might also contribute to elevated body image concerns among gay men [44,45].* *This is in contrast to findings regarding the lesbian community which seems to act as a protective factor in the development of body dissatisfaction and appearance-related concerns [50,51]” (p. 9, l. 182). We sincerely hope that this compromise meets your and the APA’s request.*

Methods
7. Continuing from my above comment, the description of the IGCS seems again to be limited to only men, but in fact, interactions with other genders within the community would also appear to count toward this scale. Please rephrase to ensure accuracy.

*As stated above, the IGCS specifically addresses engagement with the gay community (not the lesbian or any other sexual minority community). This is even more apparent in the German translation of the scale. Possible interactions with other genders are only mentioned in one item (“How often do you attend any gay or lesbian organizational activities, such as meetings, fund-raisers, political activities, etc.?”), presumably as those events are usually not restricted to the gay community but to the LGBTQ+ community as a whole.* *All other items specifically mention involvement with the gay community. To emphasize the specificity of the scale, we would like to maintain our description.*

8. Table 3 still has HOM and HEM as abbreviations.

*Thanks for making us aware of that. We changed HOM and HEM to Gay men and Heterosexual men and apologize for the mistake.*

Discussion
9. On page 32, lines 656-661, the authors argue from the place that being in a relationship means that gay men are not looking for partners. This is an untenable assumption given that polyamorous (consensual non-monogamous) relationships exist and are considered by participants to be committed. I suggest that the authors rephrase to such that men in committed relationships may be less concerned with attracting new partners.

*Thank you for raising this point. We happily included this aspect in the discussion: “Lastly, it was argued that intracommunity pressure to conform to a certain body ideal stems from the wish to attract other members of the community for sexual and social relationships [44]. However, 42% of gay men in our study were in a committed relationship, which was a similar quantity as in heterosexual men. This may have lessened the pressure from the gay community on our gay sample to stay attractive since men in committed relationships may be less concerned with attracting new partners.” (p. 43, l. 830)*

10. The phrase “…but the internal consistencies of the translated measures merely differ from those of the originals” is confusing. Maybe rephrase to “…but the internal consistencies of the translated measures are similar to the original validation studies.”

*That is indeed a more intelligible phrase, thank you for the suggestion. We were happy to exchange the phrase in our manuscript (“We did not conduct a comprehensive validation process of the translated measures, but the but the internal consistencies of the translated measures are similar to the original validation studies.”, p. 44, l. 853).*
